# Effects of cell therapy on seizures in animal models of epilepsy: Systematic review and meta‐analysis

**DOI:** 10.1111/epi.18633

**Published:** 2025-09-19

**Authors:** Afaf S. Altalhi, Muhammad S. Javaid, Nigel C. Jones, Kim L. Powell, Patrick Kwan, Terence J. O'Brien, Ana Antonic‐Baker

**Affiliations:** ^1^ Department of Neurosciences, Faculty of Medicine, Nursing and Health Sciences, School of Translational Medicine Monash University Melbourne Victoria Australia; ^2^ Department of Biotechnology, Faculty of Science Taif University Taif Saudi Arabia; ^3^ Department of Neurology Alfred Health Melbourne Victoria Australia; ^4^ Department of Neurology Royal Melbourne Hospital Melbourne Victoria Australia

**Keywords:** animal model, cell therapy, cell transplantation, epilepsy, seizures, stem cells

## Abstract

This study was undertaken to systematically evaluate the efficacy of cell therapy in reducing seizures in animal models of chronic epilepsy. Three databases, Ovid MEDLINE, Ovid Embase, and Web of Science, were searched using predetermined eligibility criteria. The relevant preclinical controlled studies were included for review and meta‐analysis using a random‐effects model to calculate summary estimates of the effect size (percentage reduction in seizures). The degree of heterogeneity among the included studies was assessed using the *I* ^2^ statistic. Subgroup meta‐analysis and meta‐regression were performed to further elucidate the sources of heterogeneity. Thirty published studies met the eligibility criteria, including a total of 1306 animals. The majority of studies used kainic acid and pilocarpine status epilepticus models of mesial temporal lobe epilepsy (MTLE). The random effects model revealed an overall reduction in seizure frequency of 54.8% (95% confidence interval = 48.0558–61.5455) compared to the control, and the heterogeneity was 87.1% among the included studies. The meta‐regression revealed that seven study characteristics significantly accounted for the between‐study heterogeneity. They can be grouped into three broad categories: epilepsy‐specific, animal‐specific, and cell transplantation‐specific. The greatest seizure reduction was observed in the post‐kainic acid status epilepticus model of chronic MTLE, when the cells were delivered intravenously and when the seizure reduction was measured as seizure frequency. Embryonic stem cell transplantation showed the greatest efficacy in reducing seizures. Cell transplantation shows favorable efficacy as a treatment that can reduce seizure recurrence in chronic animal models of epilepsy. High heterogeneity between studies reflects the diverse methodologies employed in preclinical research on cell therapy for epilepsy. Despite these encouraging findings, the high risk of publication bias and variability in study design emphasize the need for further robust preclinical studies to confirm these reported outcomes.

**Key points**

- Our meta‐analysis revealed that the overall reduction in seizures following cell transplantation in animal models of chronic epilepsy was 54.8% (95% CI = 48.1–61.5).
- The highest seizure reduction was observed in the chronic post‐kainic acid status epilepticus model of TLE when the cells were delivered intravenously, and when the seizure reduction was measured as seizure frequency.
- Most studies have used MGE progenitors, which show moderate seizure reduction.

## 1 INTRODUCTION

Recent advances in regenerative medicine have provided opportunities to develop cell‐based therapies as a potential disease‐modifying treatment for drug‐resistant epilepsy. Cell transplantation is a promising therapeutic approach for a wide variety of neurological diseases.^1^, ^2^ The rationale for this approach is that stem cells have the ability to differentiate into different cell types to replace damaged or lost cells in regions of the nervous system that are implicated in acquired epilepsies, such as in the hippocampus in mesial temporal lobe epilepsy (MTLE). A significant loss of specific types of inhibitory γ‐aminobutyric acidergic (GABAergic) interneurons including somatostatin and parvalbumin are observed in both chronic animal models of epilepsy and hippocampus resected from patients with chronic MTLE, potentially altering the balance between excitation and inhibition in epileptogenic neuronal networks.^3^, ^4^ Pallial GABAergic interneurons, primarily derived from the medial ganglionic eminence (MGE), are widely recognized as the main source of inhibition in the neocortex and hippocampus. Replacing these lost GABAergic interneurons with stem cell‐based transplantation may provide an innovative strategy to improve both seizure, cognitive, and neuropsychiatric outcomes in patients with MTLE, with the latter two common epilepsy comorbidities also likely contributed to by the cell loss. Reflecting the potential of this approach, a commercially sponsored first‐in‐human commercial phase I/II clinical trial is currently underway to assess the effects of MGE‐derived GABAergic interneurons in patients with drug‐resistant MTLE (Clinical Trials number: NCT05135091).^5^, ^6^ Additionally, several small, uncontrolled, open‐labeled, clinical studies have also reported the potential of cell transplantation for epilepsy, particularly in drug resistance.^7^, ^8^, ^9^, ^10^ However, further research is required to determine the safety and effectiveness of this approach in humans and to optimize cell types, sites of transplantation, and other critical parameters.

To inform the optimal translation of this approach into clinical trials, a systematic assessment of the quality and outcomes of relevant preclinical studies is necessary. Therefore, this systematic review and meta‐analysis aimed to (1) derive a summary estimate of the efficacy of cell transplantation in animal models of epilepsy, (2) ascertain the conditions under which animal experiments demonstrate greatest efficacy, and (3) determine any effect of study quality on reported efficacy. Our primary research question was: Does cellular therapy reduce seizure frequency and duration in preclinical models of epilepsy?

## 2 MATERIALS AND METHODS

### 2.1 Protocol approval and registration

The protocol for this systematic review was written in accordance with the PRISMA‐P (Preferred Reporting Items for Systematic Review and Meta‐Analysis Protocols) guidelines^11^ through a discussion with our scientific research team comprised of clinicians (P.K. and T.J.O.) and translational scientists (M.S.J., K.L.P., N.C.J., and A.A.‐B.). The completed protocol was formally registered in PROSPERO (Prospective Register of Systematic Reviews; CRD42018103628) and published in a peer‐reviewed journal.^12^

### 2.2 Definitions

We define a publication as a distinct piece of work, including abstracts, and each publication may present data from multiple experiments. Each experiment may report outcomes across various experimental groups (for instance, the efficacy of different types of cells in treatment or number of cells transplanted). We define these as independent comparisons and extract data accordingly, adjusting the weighting in the meta‐analysis to account for the number of treatment groups associated with each control group.

### 2.3 Identification of relevant studies

Using predetermined inclusion and exclusion criteria, we identified all relevant publications by searching three electronic databases: Ovid MEDLINE, Ovid Embase, and Web of Science. The initial search was performed in March 2019 and updated in May 2024. We used the following expanded search terms: for epilepsy, “epilepsy” or “temporal lobe epilepsy” or “TLE” or “seizure” or “epileptogenesis” or “spontaneous recurrent seizures” or “SRS” or “genetic generalised epilepsy” or “GGE” or “frontal lobe epilepsy” or “FLE” or “photosensitive epilepsy”; for stem cells, “stem cells” or “stem” or “hematopoietic” or “mesenchymal”; for cell therapy, “cell therapy” or “cell transplantation”, with the search limited to in vivo animal studies (complete search string for Ovid MEDLINE can be found in Table S1).

### 2.4 Inclusion and exclusion criteria

We included controlled studies that reported the efficacy of cell transplantation in animal models of epilepsy where the outcome was expressed as a change in seizures, where we could determine the number of animals in each group and the mean effect size and its variance. We limited our search to kainic acid, pilocarpine, and genetic epilepsy models and excluded kindling, because the latter model does not routinely exhibit spontaneous seizures. We excluded studies that did not include vehicle treatment; studies where the intervention did not involve cell‐based therapy; studies with models that did not examine epilepsy as the primary outcome; human studies; studies that did not use electroencephalography (EEG) to measure the primary outcome (seizures); reviews; editorials; and books. In this systematic review, we only included studies that used EEG as a method of seizure detection. This approach was taken to reduce methodological variability, and to ensure consistency and accuracy in seizure quantification across studies.

### 2.5 Outcomes measured

The outcomes include seizure duration, seizure frequency, time in seizure, and total number of seizures measured by EEG to assess changes in seizures following cell transplantation.

### 2.6 Publication selection

We used Covidence software to manage all the retrieved publications. Once all the duplicates were removed, we screened the remaining studies for title and abstract, followed by the full text. Two authors (A.F. and M.S.J.) independently reviewed the title and abstract of the included studies. The same two authors also independently screened the full text of each publication. A third reviewer (A.A.) resolved any disagreements that arose between the two reviewers. Data extraction was carried out on publications that met the eligibility criteria.

### 2.7 Data extraction

Data extraction was done by A.A., A.F., and N.D. using a predefined data extraction Excel form. Extracted data included publication details (publication year, title, and first author) and study characteristics related to the intervention (animal model, epilepsy model, cell tissue source, cell dose, time of administration, single or multiple doses, species of cell source, route of administration, targeted area, time of assessment). Favorable outcome was defined as a reduction in seizure duration, seizure frequency, time in seizure, and total number of seizures as primary outcomes. Additionally, mean ± variance for seizure reduction was extracted from each cohort exposed to the intervention (control or cell‐based therapy). The data were extracted from tables and graphs using a digital pixel ruler.

### 2.8 Evaluation of risk of bias

To assess the methodological quality of the included animal studies, we applied the SYRCLE Risk of Bias (RoB) tool, which is specifically adapted from the Cochrane RoB tool for use in animal intervention studies.^13^ The SYRCLE tool evaluates 10 domains of bias including selection bias, performance bias, detection bias, attrition bias, reporting bias, and other sources of bias. Two reviewers independently assessed each study, and discrepancies were resolved through consensus. We also referred to best‐practice guidance as outlined by Ineichen et al.^14^

### 2.9 Data analysis

For each independent comparison, we calculated a normalized effect size as a percentage improvement/worsening of outcome after cell intervention in the treatment group compared to the vehicle. This was done as follows. For each individual comparison, we calculated a normalized effect size (ESi; normalized mean difference) as the percentage improvement or worsening of outcomes in a treatment group using the following formula:

$$\mathrm{ESi}=100\%\times \left(\mathrm{Xc}–\mathrm{Xrx}\right)/\mathrm{Xc}$$

where Xc and Xrx are the mean reported outcomes in the control and treatment group, respectively.^15^

To address potential nonindependence of data within studies reporting multiple experimental groups, we evenly divided shared control group data across the relevant treatment arms. For studies reporting outcomes at multiple time points, only the final time point was included in the analysis to maintain consistency and reduce within‐study dependence. This approach was taken to minimize bias from double‐counting and to approximate independence where dependency‐adjusted meta‐analytic models were not feasible due to limited reporting.

We then used DerSimonian and Laird random effects weighted mean difference to calculate summary estimates of the effect size (percentage improvement) and its 95% confidence intervals (CIs). We assessed the degree of heterogeneity among the included studies using *I* ^2^ to calculate the overall percentage of the variation in effect estimates caused by heterogeneity rather than random error. We performed subgroup meta‐analysis and meta‐regression to further elucidate the sources of heterogeneity with a significance level of *p* < .05. This was done by calculating the adjusted *R* ^2^ values to determine the percentage of total heterogeneity that can be explained by the model.^16^ Then, we stratified the analysis according to animal species/strain/age, epilepsy model, cell tissue source, cell type, cell dose, species of cell source, route of administration, targeted area, time assessment, dosage regimen, and type of outcome measured. The data are presented in bar graphs; the height of each bar represents the mean effect size for each category, whereas the width of the bar represents the number of animals used to assess specific comparison; the wider the bar, the more animals were used. Vertical error bars represent the 95% CIs for the individual estimates. Shaded horizontal bars illustrate the global estimate of effect size with its 95% CIs. We sought out evidence of publication bias using a funnel plot and Egger regression. We performed all analysis using the Camarades meta‐analysis tool (https://camarades.shinyapps.io/meta‐analysis‐app/).

## 3 RESULTS

### 3.1 Study selection and characteristics

**FIGURE 1:**
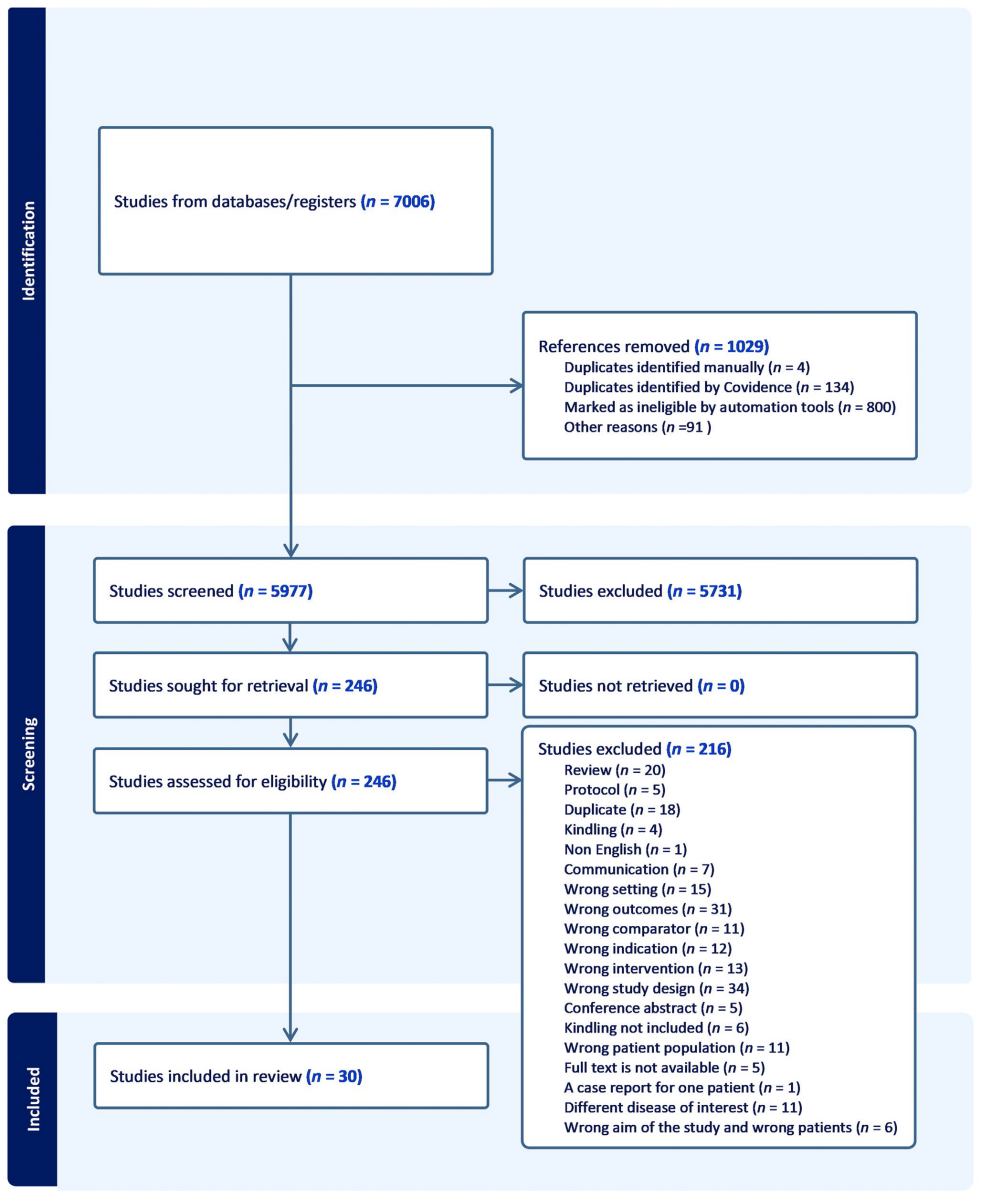
PRISMA (Preferred Reporting Items for Systematic Reviews and Meta‐Analyses) flowchart of included studies publications.

We identified a total of 7006 publications through electronic search. After removing 1029 duplicates, the remaining 5977 were initially screened by title and abstract. Two hundred forty‐five full‐text publications met our prospective inclusion criteria, resulting in the exclusion of 5731 publications (Figure 1). Ultimately, we included 30 publications in the review, with 75 independent comparisons studying a total of 1306 animals. The first report meeting criteria on the use of cell therapy in animal models of epilepsy was in 2004, investigating the antiepileptogenic potential of neural stem cells (NSCs) in the adult status epilepticus rat model.^17^ The results of this study indicated that NSCs have the potential to differentiate into inhibitory interneurons and reduce neural excitability. A further 29 publications have been published since then, contributing to this field, testing efficacy across a range of different cell types. A variety of different cell types have been employed, including MGE progenitors (eight publications), NSCs (seven publications), and bone marrow‐derived mesenchymal stem cells (seven publications). Most of the cells were obtained from primary tissue (20 publications), whereas the remaining 11 publications used cell lines. Cell dose ranged from 10 000 to 10 000 000 cells, either as a single dose (14 publications) or as multiple doses (15 publications). The cells were transplanted either intravenously or as intracranial injections targeting amygdala, substantia nigra, neocortex, cortex, and hippocampus. Animal models encompassed both rats (10 publications) and mice (21 publications), along with various strains. Three different methods were used to induce epilepsy in these studies. Pilocarpine was the most common (16 publications), followed by kainic acid (12 publications), whereas genetically modified models were less commonly used (three publications). The seizure frequency was the most common assessment of seizure outcome (21 publications) followed by seizure duration (17 publications). These seizure outcomes were assessed either in the acute phase (within 30 days) or at the chronic stage (>300 days).

### 3.2 Internal validity and publication bias

**FIGURE 2:**
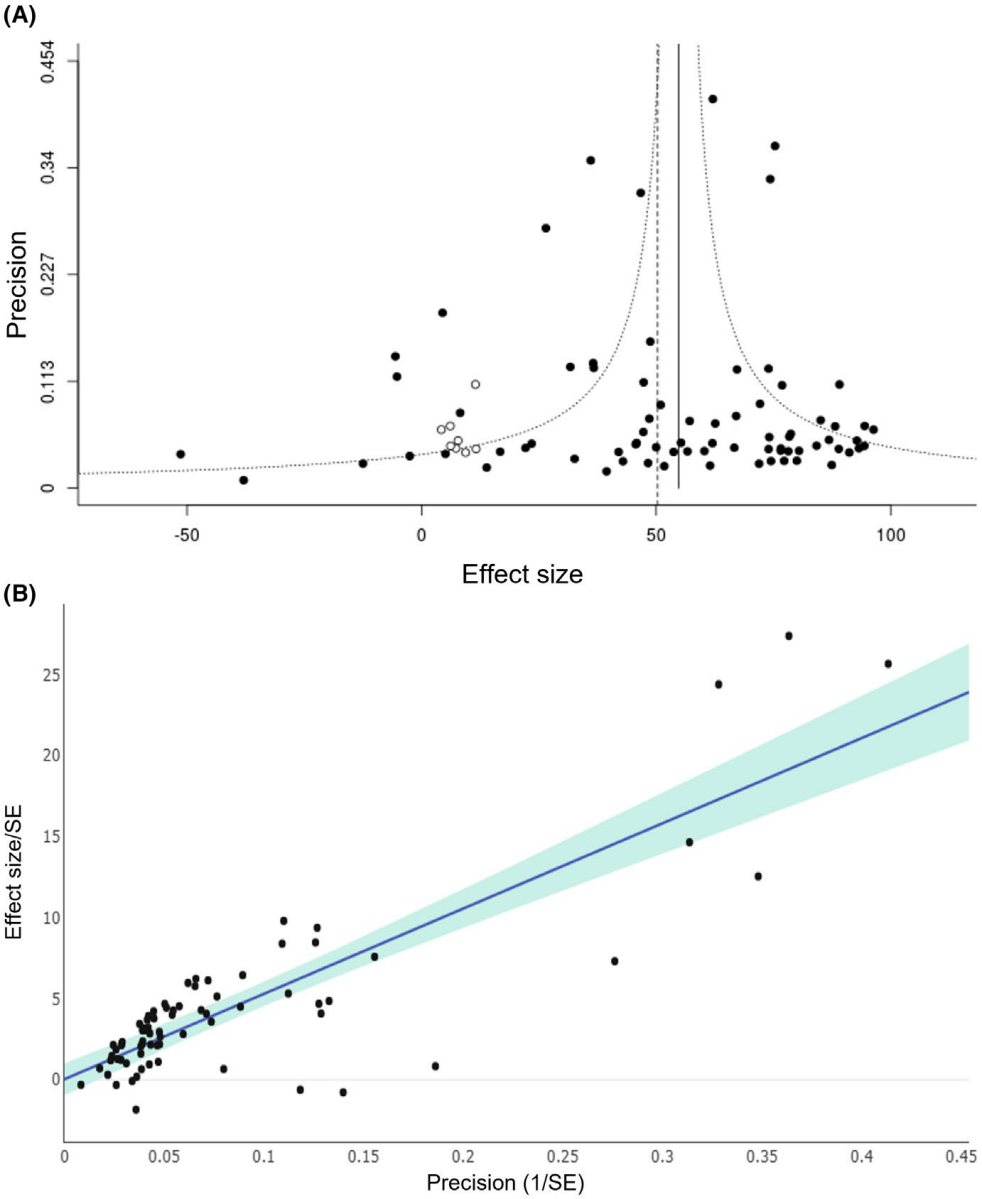
Publication bias evaluation using funnel plot (A), Egger regression (B). The funnel plot shows the data in black, and the additional missing studies are identified by trim and fill with hollow circles. SE, standard error.

All 30 included publications were assessed for the risk of bias. The study quality checklist elements, and the proportion of publications meeting these checklists, are described in Table 1. Overall, the quality of reporting was variable. Although most studies reported the use of randomization and blinding, they did not provide sufficient detail on how these procedures were implemented. In addition, the majority of studies did not explicitly state whether incomplete outcome data were addressed, rendering the risk of attrition bias unclear. Few studies described blinding of outcome assessors or allocation concealment. These findings highlight potential sources of bias that should be considered when interpreting the results. Only one of the included publications reported all of these measures designed for minimizing bias. Asymmetry in the funnel plot (Figure 2A) was detected, which is consistent with the presence of publication bias, and this was confirmed with Egger regression (Figure 2B). Trim and fill statistics identified nine missing studies (depicted in hollow circles), and a reduction in effect size from 54.8% (95% CI = 48.0558–61.5455) to 54.2% (95% CI = 48.7–59.7).

**TABLE 1:** Risk of bias assessment.

Surname	Was the allocation sequence adequately generated and applied?	Were the groups similar at baseline or were they adjusted for confounders in the analysis?	Was the allocation to the different groups adequately concealed during the experiment?	Were the animals randomly housed during the experiment?	Were the caregivers and/or investigators blinded to which intervention each animal received during the experiment?	Were animals selected at random for outcome assessment?	Was the outcome assessor blinded?	Were incomplete outcome data adequately addressed?	Are reports of the study free of selective outcome reporting?	Was the study apparently free of other problems that could result in high risk of bias?
Abdanipour (2011)	No	Yes	Unclear	Unclear	Yes	Unclear	Yes	Yes	Yes	Yes
Baraban (2009)	No	Yes	No	No	No	No	No	Unclear	Yes	Yes
Casalia (2017)	No	Yes	Yes	No	Yes	No	Yes	Unclear	Yes	Yes
Chu (2004)	Unclear	Yes	No	No	No	Yes	No	Yes	Yes	Yes
Costa‐Ferro (2010)	No	Yes	Unclear	No	Yes	No	Yes	Yes	Yes	Yes
Cunningham (2014)	Unclear	Yes	Unclear	No	Yes	Yes	Yes	Yes	Yes	Yes
Fukumura (2018)	No	Yes	No	No	No	Yes	No	Yes	Yes	Yes
Hammad (2015)	No	Yes	No	No	No	No	No	Yes	Yes	Yes
Hattiangady (2008)	No	Yes	No	No	Yes	No	Yes	Yes	Yes	Yes
Huang (2016)	No	Yes	No	No	No	Unclear	No	Yes	Yes	Yes
Hunt (2013)	Unclear	Yes	Unclear	No	Yes	Yes	Yes	Unclear	Yes	Yes
Lee (2014)	Unclear	Yes	Unclear	Unclear	Yes	Yes	Yes	Unclear	Yes	Yes
Li (2009)	No	Yes	No	No	No	No	No	Yes	Yes	Yes
Rao (2007)	No	Yes	No	No	No	No	No	Unclear	Yes	Yes
Romariz (2017)	No	Yes	No	No	No	No	No	Unclear	Yes	Yes
Venturin (2011)	No	Yes	No	No	No	No	No	Yes	Yes	Yes
Upadhya (2019)	Unclear	Yes	Yes	No	Yes	Yes	Yes	Yes	Yes	Yes
Xu (2019)	Unclear	Yes	Unclear	No	Unclear	No	Yes	Yes	Yes	Yes
Jing (2009)	No		No	No	No	No	No	Unclear	Yes	Yes
Du (2019)	Unclear	Yes	No	No	No	Yes	No	Yes	Yes	Yes
Hattiangady (2020)	Yes	Yes	Yes	Unclear	Yes	No	Yes	Yes	Yes	Yes
Lentini (2021)	Unclear	Yes	No	Unclear	No	Yes	No	Unclear	Yes	Yes
Waldau (2010)	No	No	No	No	No	No	No	Yes	Yes	Yes
Wang (2021)	Unclear	Yes	No	No	No	Yes	No	Yes	Yes	Yes
Zhu (2023)	Unclear	Yes	Unclear	No	Yes	Yes	Yes	Yes	Yes	Yes
Anderson (2018)	No	Yes	No	No	No	No	No	Yes	Yes	Yes
Castillo (2010)	No	Yes	No	No	No	No	No	Yes	Yes	Yes
Bershteyn (2023)	Yes	Yes	Yes	No	Yes	Yes	Yes	Yes	Yes	Yes
Costa‐Ferro (2014)	Unclear	Yes	No	No	No	Yes	No	Yes	Yes	Yes
Costa‐Ferro (2012)	Unclear	Yes	Yes	Yes	Yes	Yes	Yes	Yes	Yes	Yes

### 3.3 Impact of various aspects of study design

**FIGURE 3:**
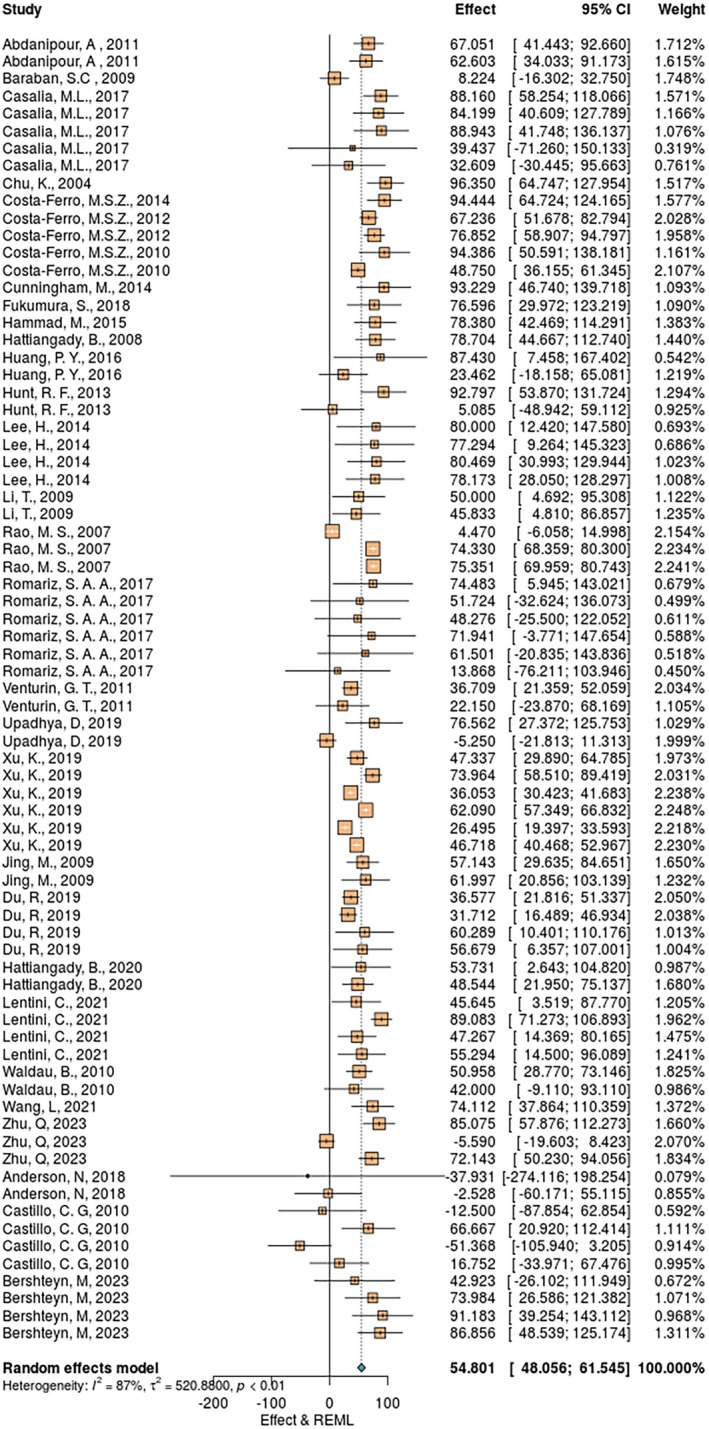
Summary of the independent comparisons effect size and heterogeneity. Each square represents the effect size (seizure reduction) of every individual comparison, and the horizontal lines represent the 95% confidence intervals (CI). The blue diamond at the bottom indicates the overall pooled effect size. REML, random effect model.

Across all 30 publications, the overall reduction in seizures following cell transplantation was 54.8% (95% CI = 48.0558–61.5455). A substantial heterogeneity was identified among the included studies (*I* ^2^ = 87.1%, *p* < .0001; Figure 3). The meta‐regression revealed that seven study characteristics significantly accounted for the observed between‐study heterogeneity (Table S3). They can be grouped into three broad categories: epilepsy‐specific, animal‐specific, and cell transplantation‐specific.

#### 3.3.1 Epilepsy‐related characteristics

**FIGURE 4:**
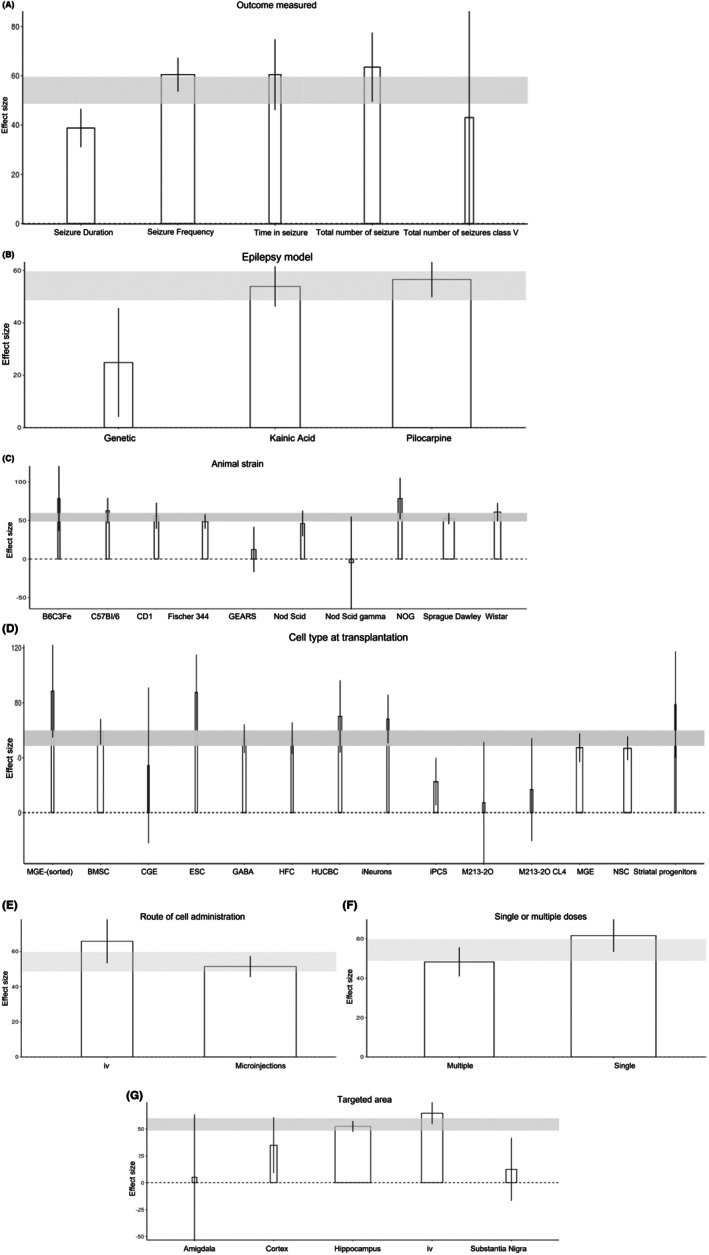
Study characteristics that accounted for heterogeneity of the effect size of seizure reduction. Bars represent the mean effect size for each category; vertical error bars represent the 95% confidence intervals for the individual estimates. Shaded horizontal bars illustrate the global estimate of effect size, and the width of each bar represents the number of animals participating in that specific comparison. (A) Type of outcome measured, (B) epilepsy model, (C) animal strain, (D) cell type transplanted, (E) target area, (F) route of cell administration, (G) number of treatments (single/multiple). BMSC, bone marrow‐derived mesenchymal stem cell; CGE, caudal ganglionic eminence; ESC, embryonic stem cell; GABA, γ‐aminobutyric acid; HFC, human fibroblast cell; HUCBC, human umbilical cord blood cell; iPCS, induced pluripotent stem cell; iv, intravenous; MGE, medial ganglionic eminence; NSC, neural stem cell.

Our analysis identified that the outcome measures used to assess seizure reduction (Figure 4A) and the models employed to induce epilepsy (Figure 4B) contributed significantly to the between‐study heterogeneity, with *R* ^2^ values of 48.17% and 32.46%, respectively. Among the assessed outcomes, the greatest effect of cell transplantation was observed in studies measuring time spent in seizure, demonstrating a mean decrease of 62.69% (95% CI = 41.25–83.28) compared to vehicle control. However, this outcome was evaluated in only four independent comparisons involving a total of 80 animals. The most frequently reported outcome measure was a reduction in seizure frequency, accounting for >50% of included studies, with 33 independent comparisons and a total of 618 animals.

Regarding epilepsy induction methods, kainic acid and pilocarpine administration were the most used, encompassing 69 independent comparisons and 1194 animals. Among these, the kainic acid model demonstrated the most significant reduction in seizures following cell transplantation, with a mean decrease of 53.67% (95% CI = 46.29–61.05) compared to vehicle control.

#### 3.3.2 Animal‐related study characteristics

Whereas the animal species did not appear to significantly influence the observed heterogeneity, the animal strain accounted for *R* ^2^ = 56.34% (Figure 4C). The most significant seizure reduction was observed in the immunodeficient (NOG) mouse model, demonstrating a mean decrease in seizures of 79.04% (95% CI = 54.83–103.24) compared to the vehicle‐treated control. This strain was, however, only used in one publication, which included four comparisons and 62 animals.^5^ In addition to this publication, three more publications used immunodeficient mice, showing a wide range of seizure reduction, ranging from 58.78% to −4.52%.

Among rat stains, Sprague Dawley and Wistar were the most commonly used, encompassing 35 independent comparisons and 723 animals. These strains showed a moderate reduction in seizures, with a mean reduction of 47.66% (95% CI = 45.04–50.27) and 62.49% (95% CI = 42.24–82.73), respectively. Notably, both strains are wielded type, and immunosuppression was not used to facilitate cell transplantation.

#### 3.3.3 Cell transplantation‐related study characteristics

Study characteristics related to cell transplantation accounted for the largest source of between‐study heterogeneity, with the type of transplanted cells contributing the most (*R* ^2^ = 68.68%; Figure 4D). Among the cell types evaluated, human pallial MGE‐type GABAergic interneuron cells and embryonic stem cells showed the most significant reduction in seizures, with a mean reduction of 88.38% (95% CI = 57.55–119.2) and 87.15% (95% CI = 63.68–110.63), respectively, compared to the vehicle control. These cells were, however, only assessed in four independent comparisons among 73 animals. Approximately half of the comparisons used either MGE (19 independent comparisons, 247 animals) or neuronal stem cells (14 comparisons, 352 animals).

The hippocampus was the most frequently targeted site for transplantation, encompassing 57 independent comparisons and 868 animals (Figure 4E). Transplantation into the hippocampus resulted in a mean seizure reduction of 52.58% (95% CI = 50.56–54.59) compared to vehicle controls.

Administering the cells intravenously via infusion or microinjection (Figure 4F) showed an approximately similar improvement in the outcome, by 66.13% (95% CI = 52.54–79.72) and 51.33% (95% CI = 45.47–57.19), respectively. Interestingly, administering cells as a single dose injection is associated with greater seizure reduction, with a mean reduction of 57.67% (95% CI = 53.01–62.34), compared to multiple injections, with a mean reduction of 49.09% (95% CI = 37.98–60.19; Figure 4G).

There was no detectable contribution to the heterogeneity by animal model, cell source, cell dose, time of administration, or time of assessment.

## 4 DISCUSSION

Cell transplantation has emerged as a promising approach for disease‐modifying therapy for epilepsy, to effectively reduce seizure occurrence in a sustained manner through various potential mechanisms that can reduce the epileptogenicity of neuronal networks such as replacement of lost cells, restoring cells, reorganizing synaptic connection, and regulation of neurotransmitters and neurotrophic factors.^18^, ^19^, ^20^, ^21^ Several small open‐label clinical studies have supported the therapeutic potential of the transplantation of a range of different types of stem cells to control seizures.^7^, ^8^, ^9^, ^10^ A recently published systematic review^22^ reported that stem cell therapy is a potentially effective treatment option for patients with drug‐resistant epilepsy; however, the current data available in clinical settings are limited and of low quality.

In the current work, we summarize the data from 30 preclinical publications identified through our systematic review that assessed the efficacy of cell transplantation in reducing seizures in chronic animal models of epilepsy. The meta‐analysis revealed an overall 54% reduction in seizure occurrence following cell transplantation. However, substantial heterogeneity was noted across studies (*I* ^2^ = 87%), highlighting the variability in experimental design and methodology in the preclinical epilepsy cell therapy literature.

Our findings suggest that the highest seizure reduction was observed in the chronic post‐kainic acid status epilepticus models of MTLE, when the cells were delivered intravenously, and when the seizure reduction was measured as a reduction in seizure frequency. Interestingly, cell transplantation did not significantly reduce seizures in genetic models of epilepsy. The lack of significant efficacy in genetic models may reflect that stem cell transplantation therapies are more effective in lesional focal epilepsy associated with cell loss, which is not generally associated with the genetic epilepsies in animal models or patients, rather than a broader antiepileptic effect. This reinforces the potential therapeutic utility of cell transplantation approaches particularly in acquired, “lesional” epilepsies such as MTLE, traumatic brain injury, or stroke, and suggests a need for careful patient stratification in clinical trials.

Most studies used MGE progenitors, which show moderate seizure reduction. The antiepileptic mechanism of transplanted cells is not fully understood, and there is a possibility that this effect could be due to paracrine mechanisms that incorporate local cellular migration and the subsequent release of neurotrophic factors. For example, transplantation of MGE progenitor cells into the hippocampal region of epileptic rodents resulted in a significant reduction in seizure frequency and normalized behavior, possibly through the release of inhibitory neurotransmitters and trophic factors that promote neuronal health and synaptic integration.^23^, ^24^ Furthermore, human umbilical cord blood‐derived mononuclear cells have been associated with neuroprotective effects via a similar mechanism, promoting neurogenesis and reducing neuronal injury in models of epilepsy.^25^ These mechanisms would be complementary to the direct effects of the integrated cells themselves, and further research is required to elucidate the precise mechanisms.

Factors such as cell dose, time of stem cell administration, and time of assessment of outcome appear to be less important. The duration of the therapeutic effect following cell transplantation remains an important consideration. In this meta‐analysis, we included only the final time point assessed in each study, which limited our ability to analyze the time course of treatment effects. However, as all studies reported outcomes at their latest follow‐up (up to 6 months posttransplantation in some studies), the effects observed are unlikely to be transient. This suggests that cell transplantation may have a sustained therapeutic impact. Nonetheless, future studies with extended longitudinal follow‐up are needed to more fully characterize the durability and progression of treatment effects over time. Notably, one of the most unexpected findings was that a very small proportion of studies utilized immunodeficient animals or implemented immunosuppression. Despite this, the absence of immunosuppression did not appear to negatively impact the therapeutic outcomes of cell transplantation, suggesting that graft survival and functional integration may be achievable even in the absence of immune modulation, at least in rodents. This finding warrants further investigation, particularly in the context of translating cell‐based therapies to clinical applications, where minimizing the need for immunosuppressive therapy would be a great advantage for patients.

Although these findings offer valuable guidance for designing clinical trials, there remains a limited emphasis on measures to improve the internal and subsequent risk of publication bias. Our risk of bias assessment using the SYRCLE tool revealed several methodological limitations in the included studies, particularly concerning incomplete outcome data and selective reporting. That most studies did not clearly report whether data were missing or how this was handled introduces uncertainty regarding attrition bias. Likewise, selective reporting bias was difficult to evaluate in the absence of prespecified protocols. These limitations underscore the need for improved transparency and methodological rigor in preclinical animal research. The high risk of publication bias observed here could be attributed to the likelihood that many studies, particularly with unfavorable outcomes, remain unpublished.

Of particular note is the recently published study by Bershteyn and colleagues, which provided the key preclinical evidence used to inform the first‐in‐human commercial phase I/II clinical trial of stem cell transplantation therapy for epilepsy, specifically unilateral MTLE, currently underway and sponsored by Neurona.^5^ The preclinical study utilized a post‐kainic acid‐induced status epilepticus epilepsy model of chronic MTLE, in an NOG immunodeficient mice strain, transplanting modified human MGE progenitors into the hippocampus. Notably, it was the only study to achieve the maximum quality assessment score of seven, indicating the highest methodological rigor. The study reported an estimated seizure reduction of 88%, representing one of the most significant effects observed among the included studies.

Our review approach has some limitations. The nature of a systematic review is an observational rather than experimental assessment of literature, so we can only report associations rather than causation. Although we designed our search strategy to be comprehensive, some studies may have been inadvertently missed. However, our study likely encompasses the majority of reports in this field, representing the most thorough review to date on the use of stem cells in epilepsy. Another limitation of this analysis is the lack of standardized reporting on behavioral seizure types across studies (e.g., focal vs. generalized, electrographic vs. motor). This limited our ability to characterize them for meta‐regression, which could have provided insights into the differential efficacy of cell therapy for specific seizure types. Future studies should aim to consistently document seizure characteristics to support more detailed and informative analyses. Due to these limitations, hypotheses generated from this work require validation through rigorously designed, adequately powered head‐to‐head experiments.

Nevertheless, our analysis provides support for the promise of cell therapies as a disease‐modifying therapy approach for patients with chronic epilepsy, in particular acquired epilepsies such as MTLE. In particular, our findings show that MGE progenitors transplanted into a kainic acid post‐status epileptic animal model of chronic MTLE had a significant reduction in seizure frequency, supporting the hypothesis that the transplanted cells replace lost and damaged tissue in the epileptic zone, restoring normal brain function. This is of great relevance, as this GABAergic neuronal population is known to be most affected in patients with drug‐resistant chronic MTLE.^26^, ^27^, ^28^

## AUTHOR CONTRIBUTIONS

**Afaf S. Altalhi:** Original draft preparation (lead); writing—review and editing (equal); conceptualization (equal); investigation (equal); visualization (lead). **Muhammad S. Javaid:** Review and editing (equal); investigation (equal). **Nigel C. Jones:** Writing—review and editing (equal); investigation (equal). **Kim L. Powell:** Writing—conceptualization (equal); review and editing. **Patrick Kwan:** Writing—review and editing (equal). **Terence J. O'Brien:** Conceptualization (equal); writing—review and editing (equal); funding acquisition. **Ana Antonic‐Baker:** Conceptualization (equal); investigation (equal); writing—review and editing (equal).

## CONFLICT OF INTEREST STATEMENT

The authors declare no conflict of interest. We confirm that we have read the Journal's position on issues involved in ethical publication and affirm that this report is consistent with those guidelines.

## Supporting information


Table S1.

Table S2.

Table S3.

## Data Availability

The data supporting this study's findings are available in the supplementary material of this article.
