# Supplementary material for: Effects of cell therapy on seizures in animal models of epilepsy: Systematic review and meta‐analysis
Source: Epilepsia. 2025 Sep 19;67(1):13–26. doi: 10.1111/epi.18633 (PMC12893257; doi:10.1111/epi.18633)
Supplement: Supplementary file 1 — Table S1. [file EPI-67-13-s003.docx]

Database(s): **Ovid MEDLINE(R) and Epub Ahead of Print, In-Process, In-Data-Review & Other Non-Indexed Citations, Daily and Versions**

1946 to May 30, 2024

Search Strategy:

| **#** | **Searches** | **Results** |
| --- | --- | --- |
| 1 | ("stem cells" or "stem" or "haematopoietic" or "mesenchymal").mp. | 679583 |
| 2 | ("Epilepsy" or "Temporal lobe epilepsy" or "TLE" or "seizure" or "epileptogenesis" or "spontaneous recurrent seizures" or "SRS" or "Genetic Generalised Epilepsy" or "GGE" or "frontal lobe epilepsy" or "FLE" or "Photosensitive epilepsy").mp. | 212365 |
| 3 | ("cell therapy" or "cell transplantation"). mp. | 172204 |
| 4 | 1 and 2 and 3 | 359 |
| 5 | Limit 4 to animals | 159 |
