# Supplementary material for: Effects of cell therapy on seizures in animal models of epilepsy: Systematic review and meta‐analysis
Source: Epilepsia. 2025 Sep 19;67(1):13–26. doi: 10.1111/epi.18633 (PMC12893257; doi:10.1111/epi.18633)
Supplement: Supplementary file 2 — Table S2. [file EPI-67-13-s002.docx]

**Reference list of included papers**

1. Lentini C, d'Orange M, Marichal N, Trottmann MM, Vignoles R, Foucault L, et al. Reprogramming reactive glia into interneurons reduces chronic seizure activity in a mouse model of mesial temporal lobe epilepsy. Cell Stem Cell. 2021;28(12):2104-21.e10.

2. Lee H, Yun S, Kim IS, Lee IS, Shin JE, Park SC, et al. Human fetal brain-derived neural stem/progenitor cells grafted into the adult epileptic brain restrain seizures in rat models of temporal lobe epilepsy. PLoS One. 2014;9(8):e104092.

3. Zhu Q, Mishra A, Park JS, Liu D, Le DT, Gonzalez SZ, et al. Human cortical interneurons optimized for grafting specifically integrate, abort seizures, and display prolonged efficacy without over-inhibition. Neuron. 2023;111(6):807-23.e7.

4. Xu K, Liu F, Xu W, Liu J, Chen S, Wu G. Transplanting GABAergic Neurons Differentiated from Neural Stem Cells into Hippocampus Inhibits Seizures and Epileptiform Discharges in Pilocarpine-Induced Temporal Lobe Epilepsy Model. World Neurosurgery. 2019;128:e1-e11.

5. Wang L, Zhao Y, Pan X, Zhang Y, Lin L, Wu Y, et al. Adipose-derived stem cell transplantation improves learning and memory via releasing neurotrophins in rat model of temporal lobe epilepsy. Brain Research. 2021;1750 (no pagination)(147121).

6. Waldau B, Hattiangady B, Kuruba R, Shetty AK. Medial ganglionic eminence-derived neural stem cell grafts ease spontaneous seizures and restore GDNF expression in a rat model of chronic temporal lobe epilepsy. Stem Cells. 2010;28(7):1153-64.

7. Venturin GT, Greggio S, Marinowic DR, Zanirati G, Cammarota M, Machado DC, et al. Bone marrow mononuclear cells reduce seizure frequency and improve cognitive outcome in chronic epileptic rats. Life Sciences. 2011;89(7-8):229-34.

8. Upadhya D, Hattiangady B, Castro OW, Shuai B, Kodali M, Attaluri S, et al. Human induced pluripotent stem cell-derived MGE cell grafting after status epilepticus attenuates chronic epilepsy and comorbidities via synaptic integration. Proceedings of the National Academy of Sciences of the United States of America. 2019;116(1):287-96.

9. Romariz SA, Paiva DS, Galindo LT, Barnabe GF, Guedes VA, Borlongan CV, et al. Medial Ganglionic Eminence Cells Freshly Obtained or Expanded as Neurospheres Show Distinct Cellular and Molecular Properties in Reducing Epileptic Seizures. CNS Neuroscience & Therapeutics. 2017;23(2):127-34.

10. Rao MS, Hattiangady B, Rai KS, Shetty AK. Strategies for promoting anti-seizure effects of hippocampal fetal cells grafted into the hippocampus of rats exhibiting chronic temporal lobe epilepsy. Neurobiol Dis. 2007;27(2):117-32.

11. Li T, Ren G, Kaplan DL, Boison D. Human mesenchymal stem cell grafts engineered to release adenosine reduce chronic seizures in a mouse model of CA3-selective epileptogenesis. Epilepsy Research. 2009;84(2-3):238-41.

12. Jing M, Shingo T, Yasuhara T, Kondo A, Morimoto T, Wang F, et al. The combined therapy of intrahippocampal transplantation of adult neural stem cells and intraventricular erythropoietin-infusion ameliorates spontaneous recurrent seizures by suppression of abnormal mossy fiber sprouting. Brain Research. 2009;1295:203-17.

13. Hunt RF, Girskis KM, Rubenstein JL, Alvarez-Buylla A, Baraban SC. GABA progenitors grafted into the adult epileptic brain control seizures and abnormal behavior. Nature Neuroscience. 2013;16(6):692-7.

14. Huang PY, Shih YH, Tseng YJ, Ko TL, Fu YS, Lin YY. Xenograft of human umbilical mesenchymal stem cells from Wharton's jelly as a potential therapy for rat pilocarpine-induced epilepsy. Brain, Behavior, & Immunity. 2016;54:45-58.

15. Hattiangady B, Rao MS, Shetty AK. Grafting of striatal precursor cells into hippocampus shortly after status epilepticus restrains chronic temporal lobe epilepsy. Experimental Neurology. 2008;212(2):468-81.

16. Hattiangady B, Kuruba R, Shuai B, Grier R, Shetty AK. Hippocampal neural stem cell grafting after status epilepticus alleviates chronic epilepsy and abnormal plasticity, and maintains better memory and mood function. Aging and Disease. 2020;11(6):1374-94.

17. Hammad M, Schmidt SL, Zhang X, Bray R, Frohlich F, Ghashghaei HT. Transplantation of GABAergic Interneurons into the Neonatal Primary Visual Cortex Reduces Absence Seizures in Stargazer Mice. Cerebral Cortex. 2015;25(9):2970-9.

18. Fukumura S, Sasaki M, Kataoka-Sasaki Y, Oka S, Nakazaki M, Nagahama H, et al. Intravenous infusion of mesenchymal stem cells reduces epileptogenesis in a rat model of status epilepticus. Epilepsy Research. 2018;141:56-63.

19. Du R, Zhu X, Wu S, Zhang X, He Y, Zhang K, et al. PET imaging of metabolic changes after neural stem cells and GABA progenitor cells transplantation in a rat model of temporal lobe epilepsy. European Journal of Nuclear Medicine & Molecular Imaging. 2019;46(11):2392-7.

20. Cunningham M, Cho JH, Leung A, Savvidis G, Ahn S, Moon M, et al. hPSC-derived maturing GABAergic interneurons ameliorate seizures and abnormal behavior in epileptic mice. Cell Stem Cell. 2014;15(5):559-73.

21. Costa-Ferro ZSM, Souza BSF, Leal MMT, Kaneto CM, Azevedo CM, da Silva IC, et al. Transplantation of bone marrow mononuclear cells decreases seizure incidence, mitigates neuronal loss and modulates pro-inflammatory cytokine production in epileptic rats. Neurobiology of Disease. 2012;46(2):302-13.

22. Costa-Ferro ZSM, De Borba Cunha F, De Freitas Souza BS, Leal MMT, Da Silva AA, De Bellis Kuhn TIB, et al. Antiepileptic and neuroprotective effects of human umbilical cord blood mononuclear cells in a pilocarpine-induced epilepsy model. Cytotechnology. 2014;66(2):193-9.

23. Costa-Ferro ZS, Vitola AS, Pedroso MF, Cunha FB, Xavier LL, Machado DC, et al. Prevention of seizures and reorganization of hippocampal functions by transplantation of bone marrow cells in the acute phase of experimental epilepsy. Seizure. 2010;19(2):84-92.

24. Chu K, Kim M, Jung KH, Jeon D, Lee ST, Kim J, et al. Human neural stem cell transplantation reduces spontaneous recurrent seizures following pilocarpine-induced status epilepticus in adult rats. Brain Research. 2004;1023(2):213-21.

25. Castillo CG, Mendoza S, Saavedra J, Giordano M. Lack of effect of intranigral transplants of a GABAergic cell line on absence seizures. Epilepsy & Behavior. 2010;18(4):358-65.

26. Casalia ML, Howard MA, Baraban SC. Persistent seizure control in epileptic mice transplanted with gamma-aminobutyric acid progenitors. Annals of Neurology. 2017;82(4):530-42.

27. Bershteyn M, Broer S, Parekh M, Maury Y, Havlicek S, Kriks S, et al. Human pallial MGE-type GABAergic interneuron cell therapy for chronic focal epilepsy. Cell Stem Cell. 2023;30(10):1331-50.e11.

28. Baraban SC, Southwell DG, Estrada RC, Jones DL, Sebe JY, Alfaro-Cervello C, et al. Reduction of seizures by transplantation of cortical GABAergic interneuron precursors into Kv1.1 mutant mice. Proceedings of the National Academy of Sciences of the United States of America. 2009;106(36):15472-7.

29. Anderson NC, Van Zandt MA, Shrestha S, Lawrence DB, Gupta J, Chen CY, et al. Pluripotent stem cell-derived interneuron progenitors mature and restore memory deficits but do not suppress seizures in the epileptic mouse brain. Stem Cell Research. 2018;33:83-94.

30. Abdanipour A, Tiraihi T, Mirnajafi-Zadeh J. Improvement of the pilocarpine epilepsy model in rat using bone marrow stromal cell therapy. Neurological Research. 2011;33(6):625-32.
