# Supplementary material for: Effects of cell therapy on seizures in animal models of epilepsy: Systematic review and meta‐analysis
Source: Epilepsia. 2025 Sep 19;67(1):13–26. doi: 10.1111/epi.18633 (PMC12893257; doi:10.1111/epi.18633)
Supplement: Supplementary file 3 — Table S3. [file EPI-67-13-s001.docx]

**Table S3.** Study characteristics account for observed heterogeneity among the included studies.

|  | | | | | | | | | | | | | | | | | | | | | | | |
| --- | --- | --- | --- | --- | --- | --- | --- | --- | --- | --- | --- | --- | --- | --- | --- | --- | --- | --- | --- | --- | --- | --- | --- |
| Seizure reduction | | | |  | | **Effect size % (95% CI)** | | | | | | | | | **Number of**  **Animals** | | **Number of**  **Comparisons** | | **Adjusted R^2^** | | | | **P -value** |
| Pooled estimate | | |  | | | 54.1887 [48.6649; 59.7125] | | | | | | | | | 1306 | | 75 | |  | | | |  |
|  | | |  | | |  | | |  | | |  | | |  | |  | |  | | | |  |
| Epilepsy model | |  | | | | | |  | |  | | |  | | | 32.46% | | | | 0.0175 | | | |
|  | Pilocarpine | | | | | | 50.03[95% CI 46.44; 53.62] | | | | | | | 848 | | | 41 |  | | | | |  |
|  | Genetic | | | | | | 21.02 [95% CI 17.39; 59.43 | | | | | | | 112 | | | 6 |  | | | | |  |
|  | Kainic Acid | | | | | | 53.67[95% CI 46.29; 61.05] | | | | | | | 346 | | | 28 |  | | | | |  |
| Animal strain | | | | | | | | |  | |  | | |  | | |  | 56.34% | | | 0.0149 | | |
|  | Sprague Dawley | | | | | | 47.66 [95% CI 45.04; 50.27] | | | | | | | 539 | | | 27 |  | |  | | | |
|  | CD1 | | | | | | 57.45 [95% CI 34.24; 80.66] | | | | | | | 89 | | | 8 |  | |  | | | |
|  | Wistar | | | | | | 62.49 [95% CI 42.24; 82.73] | | | | | | | 184 | | | 7 |  | |  | | | |
|  | Nod Scid | | | | | | 58.78 [95% CI 14.5; 103.06] | | | | | | | 62 | | | 4 |  | |  | | | |
|  | B6C3Fe | | | | | | 78.38 [95% CI 42.47; 114.29] | | | | | | | 20 | | | 1 |  | |  | | | |
|  | Fischer 344 | | | | | | 49.49 [95% CI 34.91; 64.07] | | | | | | | 138 | | | 12 |  | |  | | | |
|  | C57Bl/6 | | | | | | 68.81 [95% CI 56.24; 81.39] | | | | | | | 48 | | | 6 |  | |  | | | |
|  | Nod Scid gamma | | | | | | -4.52 [95% CI 60.52; 51.48] | | | | | | | 78 | | | 2 |  | |  | | | |
|  | GEARS | | | | | | 7.72 [95% CI 40.57; 56.01] | | | | | | | 80 | | | 4 |  | |  | | | |
|  | NOG | | | | | | 79.04 [95% CI 54.83; 103.24] | | | | | | | 68 | | | 4 |  | |  | | | |
| Cell type at transplantation | | | | | | | | |  | |  | | |  | | |  | 68.68% | | < 0.0001 | | | |
|  | BMSC | | | | | | 59.12 [95% CI 49.4; 68.84] | | | | | | | 218 | | | 12 |  | |  | | | |
|  | MGE | | | | | | 47.6 [95% CI 37.08; 58.12] | | | | | | | 247 | | | 19 |  | |  | | | |
|  | CGE | | | | | | 34.28 [95% CI 20.51; 89.07] | | | | | | | 15 | | | 2 |  | |  | | | |
|  | NSC | | | | | | 36.68 [95% CI 32.77; 40.58] | | | | | | | 352 | | | 14 |  | |  | | | |
|  | HUCBC | | | | | | 67.1 [95% CI 25.97; 108.22] | | | | | | | 73 | | | 3 |  | |  | | | |
|  | ESC | | | | | | 87.15 [95% CI 63.68; 110.63] | | | | | | | 34 | | | 2 |  | |  | | | |
|  | Striatal Progenitors | | | | | | 78.7 [95% CI 44.67; 112.74] | | | | | | | 10 | | | 1 |  | | | |  | |
|  | HFC | | | | | | 51.58 | | [95% CI 5.6; 97.55] | | | | | 30 | | | 3 |  | | | |  | |
|  | GABA | | | | | | 53.94 [95% CI 43.21; 64.67] | | | | | | | 72 | | | 5 |  | | | |  | |
|  | iNeurons | | | | | | 67.91 [95% CI 49.94; 85.87] | | | | | | | 30 | | | 4 |  | | | |  | |
|  | iPCS | | | | | | 16.06 [95% CI 4.51; 27.61] | | | | | | | 106 | | | 4 |  | | | |  | |
|  | M213-2O | | | | | | 7.63 [95% CI 34.45; 49.71] | | | | | | | 40 | | | 2 |  | | | |  | |
|  | M213-2O CL4 | | | | | | 8.63 [95% CI 107.03; 124.28] | | | | | | | 40 | | | 2 |  | | | |  | |
|  | Sorted MGE-pINs | | | | | | 88.38 [95% CI 57.55; 119.21] | | | | | | | 39 | | | 2 |  | | | |  | |
| Targeted area | | | | | | |  | |  |  | | | |  | | |  | 56.00% | | | | 0.0019 | |
|  | iv | | | | | | 66.13 [95% CI 52.54; 79.72] | | | | | | | 311 | | | 11 |  | | | |  | |
|  | Cortex | | | | | | 42.03 [95% CI 26.68; 110.73] | | | | | | | 32 | | | 2 |  | | | |  | |
|  | Hippocampus | | | | | | 52.58[95% CI 50.56; 54.59] | | | | | | | 868 | | | 57 |  | | | |  | |
|  | Amygdala | | | | | | 5.08 [95% CI 48.94; 59.11] | | | | | | | 15 | | | 1 |  | | | |  | |
|  | Substantia Nigra | | | | | | 7.72 [95% CI 40.57; 56.01] | | | | | | | 80 | | | 4 |  | |  | | | |
| Route of administration | | | | | | |  | |  |  | | | |  | | |  | 8.88% | | 0.0414 | | | |
|  | iv | | | | 66.13 [95% CI 52.54; 79.72] | | | | | | | | | 311 | | | 11 |  | |  | | | |
|  | Microinjections | | | | 51.33 [95% CI 45.47; 57.19] | | | | | | | | | 995 | | | 64 |  | |  | | | |
| Single or multiple delivery | | | | | | |  | |  |  | | | |  | | |  | 2.58% | | 0.0177 | | | |
|  | Single | | | | | | 57.67 | | [95% CI 53.01; 62.34] | | | | | 782 | | | 39 |  | |  | | | |
|  | Multiple | | | | | | 49.09 | | [95% CI 37.98; 60.19] | | | | | 524 | | | 36 |  | |  | | | |
| Outcome measures | | | | | | |  | |  |  | | | |  | | |  | 48.17% | | 0.0004 | | | |
|  | Total number of seizures | | | | | | 57.3 | | [95% CI 47.34; 67.25] | | | | | 141.5 | | | 11 |  | |  | | | |
|  | Seizure Duration | | | | | | 38.98 | | [95% CI 30.61; 47.36] | | | | | 426 | | | 25 |  | |  | | | |
|  | Seizure Frequency | | | | | | 61.53 | | [95% CI 53.35; 69.71] | | | | | 618.5 | | | 33 |  | |  | | | |
|  | Time in seizure | | | | | | 62.69 | | [95% CI 42.1; 83.28] | | | | | 80 | | | 4 |  | |  | | | |
|  | Total number of seizures class V | | | | | | 32.98 [95% CI 43.74; 109.69] | | | | | | | 40 | | | 2 |  | |  | | | |
